# Contribution of genetic variants to congenital heart defects in both singleton and twin fetuses: a Chinese cohort study

**DOI:** 10.1186/s13039-023-00664-y

**Published:** 2024-01-04

**Authors:** Shaobin Lin, Shanshan Shi, Jian Lu, Zhiming He, Danlun Li, Linhuan Huang, Xuan Huang, Yi Zhou, Yanmin Luo

**Affiliations:** 1 Prenatal Diagnosis Center, Department of Obstetrics and Gynecology, The First Affiliated Hospital, Sun Yat-Sen University, 58 Zhong Shan Er Road, Guangzhou, 510080 Guangdong China; 2 Fetal Medicine Center, The First Affiliated Hospital, Jinan University, No. 613 Huangpu West Road, Guangzhou, 510630 Guangdong China; 3 Medical Genetic Center, Guangdong Women and Children Hospital, Guangzhou, Guangdong China; 4 Maternal and Children Metabolic-Genetic Key Laboratory, Guangdong Women and Children Hospital, No.521, Xingnan Road, Panyu District, Guangzhou, 511400 Guangdong China

**Keywords:** Congenital heart defect, Prenatal diagnosis, Chromosomal abnormalities, Numerical chromosomal abnormality, Copy number variant, Sequence variant, Chromosome microarray analysis, Whole-exome sequencing

## Abstract

**Background:**

The contribution of genetic variants to congenital heart defects (CHDs) has been investigated in many postnatal cohorts but described in few prenatal fetus cohorts. Overall, specific genetic variants especially copy number variants (CNVs) leading to CHDs are somewhat diverse among different prenatal cohort studies. In this study, a total of 1118 fetuses with confirmed CHDs were recruited from three units over a 5-year period, composing 961 of singleton pregnancies and 157 of twin pregnancies. We performed chromosomal microarray analysis on all cases to detect numerical chromosomal abnormalities (NCAs) and pathogenic/likely pathogenic CNVs (P/LP CNVs) and employed whole-exome sequencing for some cases without NCAs and P/LP CNVs to detect P/LP sequence variants (P/LP SVs).

**Results:**

Overall, NCAs and P/LP CNVs were identified in 17.6% (197/1118) of cases, with NCA accounting for 9.1% (102/1118) and P/LP CNV for 8.5% (95/1118). Nonisolated CHDs showed a significantly higher frequency of NCA than isolated CHD (27.3% vs. 4.4%, *p* < 0.001), but there was no significant difference in the frequency of P/LP CNVs between isolated and nonisolated CHD (11.7% vs. 7.7%). A total of 109 P/LP CNVs were identified in 95 fetuses, consisting of 97 (89.0%) de novo, 6 (5.5%) parental inherited and 6 (5.5%) with unavailable parental information. The 16p11.2 proximal BP4-BP5 deletion was detected in 0.9% (10/1118) of all cases, second only to the most common 22q11.21 proximal A-D deletion (2.1%, 23/1118). Most of the 16p11.2 deletions (8/10) detected were de novo, and were enriched in CHD cases compared with a control cohort from a previous study. Additionally, SV was identified in 12.9% (8/62) of cases without NCA and P/LP CNV, most of which were de novo with autosomal dominant inheritance.

**Conclusions:**

Our cohort study provides a deep profile of the contribution of genetic variants to CHDs in both singleton and twin fetuses; NCA and P/LP CNV contribute to 9.1% and 8.5% of CHD in fetuses, respectively. We confirmed the 16p11.2 deletion as a CHD-associated hotspot CNV, second only to the 22q11.21 deletion in frequency. Most 16p11.2 deletions detected were de novo. Additionally, P/LP SV was identified in 12.9% (8/62) of fetuses without NCA or P/LP CNV.

**Supplementary Information:**

The online version contains supplementary material available at 10.1186/s13039-023-00664-y.

## Background

Congenital heart defects (CHDs) are the most common congenital structural malformations in both Chinese and other populations [1–4]. Routine screening of fetal anatomical structures using improved ultrasound technology has partly increased prenatal detection of CHD in recent decades [3, 5]. The current scope for prenatal diagnosis of CHDs not only includes cardiovascular anatomical structure but also diagnosis or exclusion of genetic disorders, which may involve prenatally undetectable functional anomalies or neurodevelopmental disorders (NDDs). Extracardiac malformations (ECMs) and NDDs are estimated to occur in approximately 13% and 10% of patients with CHDs, respectively, with approximately 2% being caused by genetic syndromes [6, 7]. Although advances in perinatal care and medical interventions have led to drastically reduced mortality rates in neonates with CHDs, genetic disorders greatly influence the outcomes and medical management of CHD patients.

Genetic evaluation for CHDs in postnatal cohorts has been well described in recent years and contributes to most of the available genetic data associated with CHDs [8–12]. The contributions of numerical chromosomal abnormality (NCA), copy number variant (CNV) and monogenic sequence variant (SV) events to CHDs are estimated to be 13%, 10% and 12% respectively [13, 14], indicating that genetic variants play a significant role in CHD pathogenesis. Nevertheless, the frequencies of various genetic variants might be somewhat different between pre- and postnatal cohorts, mainly because prenatal cohorts include intrauterine demise, termination or selective reduction for twin fetuses as well as fetuses with many other ECMs in addition to CHDs. Although a few studies on prenatal CHD cohorts have been performed to evaluate the contribution of genetic variants [15–21], our study provides a comprehensive profile of genetic variants in a larger fetus cohort (including singleton and twin fetuses) from multiple centers in southern China.

## Results

### Cohort characteristics

**Fig. 1:**
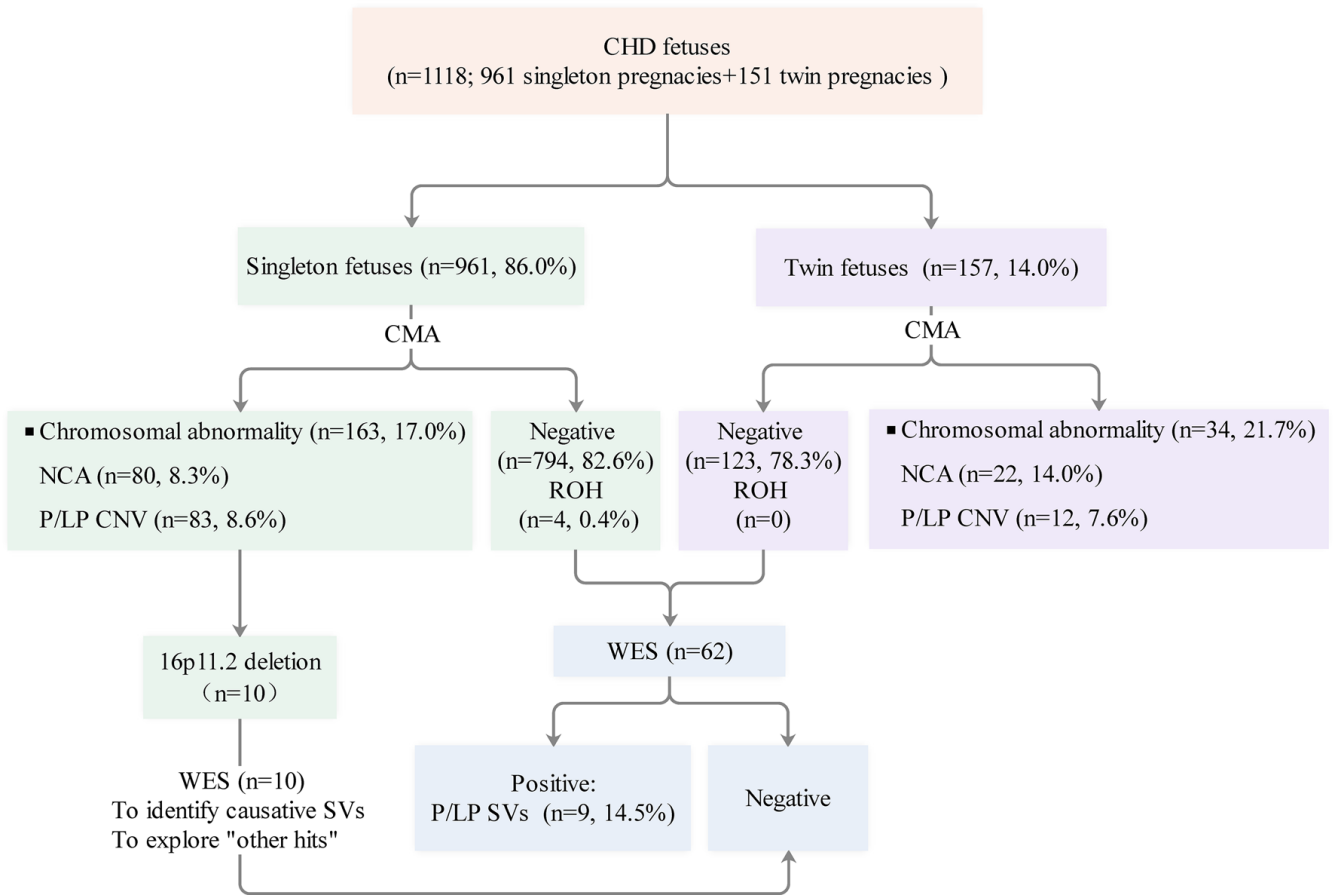
Flowchart diagram summarizing the genetic findings for singleton and twin fetuses with congenital heart defects. CHD, Congenital heart defect; CMA, Chromosomal microarray analysis; NCA, Numerical chromosomal abnormality; P/LP CNV, Pathogenic and likely pathogenic copy number variant; P/LP SV, Pathogenic and likely pathogenic sequence variant; ROH, Region of homozygosity; WES, Whole-exome sequencing. Other hits denoting cardiovascular phenotype-associated deleteriously rare variants in functionally intolerant genes

In total, 1118 fetuses including 961 from singleton pregnancies and 157 from twin pregnancies (n = 151), who underwent chromosomal microarray analysis (CMA) prenatally were included, while 61 fetuses including 55 from singleton pregnancies and 6 from twin pregnancies (n = 6), who did not undergo CMA prenatally were excluded. All parents of the 1118 fetuses were of Chinese Han ethnicity, and all parents declared their marriages were nonconsanguineous. Furthermore, prenatal CMA performed on the 1118 fetuses did not reveal large regions of homozygosity (ROHs) involving multiple chromosomes, confirming the lack of consanguineous marriage among the participating parents. Among the 151 twin pregnancies, 99 were dichorionic diamniotic, 44 monochorionic diamniotic and 8 monochorionic monoamniotic cases; both fetuses of a twin pair from 6 twin pregnancies had CHDs, and one fetus of a twin pair from 145 twin pregnancies had CHDs. CHDs were isolated in 887 fetuses (778 singleton; 109 twin) and nonisolated in 231 (183 singleton; 48 twin), with one or several ECMs (n = 192), fetal growth restriction (n = 27) or amniotic fluid volume abnormalities (n = 14). CHDs were classified into various types according to a previous study [22]. The primary cohort characteristics are summarized in Table 1. The study flowchart is illustrated in Fig. 1.

**Table 1:**
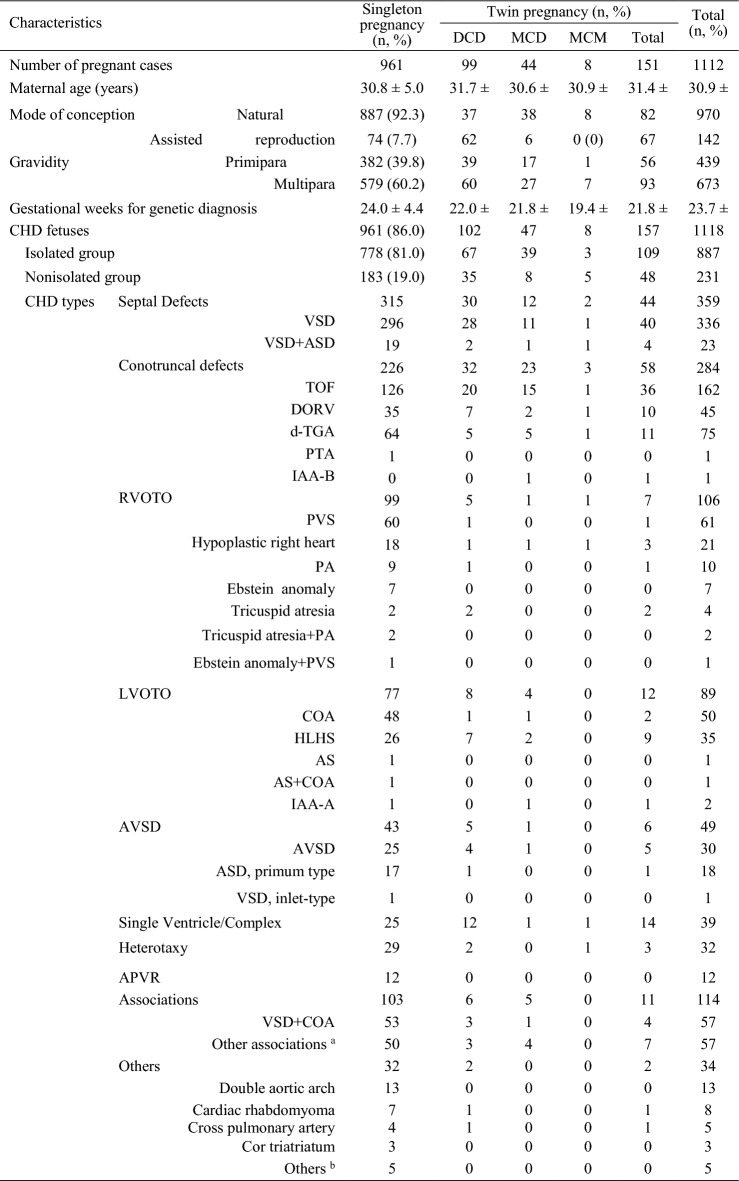
Clinical characteristics of the cases included in the present cohort

^a^Including other common, uncomplicated combinations of heart defects except for VSD + COA

^b^Including absence of pulmonary artery (n = 2), interrupted of the inferior vena cava with azygos continuation (n = 1), aortopulmo-nary septal defect (n = 1), congenital aortic sinus aneuysm (n = 1)

*AS* Aortic stenosis; *ASD* Atrial septal defect; *APVR* Anomalous-pulmonary venous return; *AVSD* Atrioventricular septal defect; *COA* Coarctation of aorta; *d-TGA* d-Transposition of great arteries; *DCDA* Dichorionic diamniotic; *DORV* Double outlet right ventricle; *HLHS* Hypoplastic left heart syndrome; *IAA* Interrupted aortic arch; *LVOTO* Left ventricular outflow tract obstruction; *MCDA* Monochorionic diamniotic; *MCMA* Monochorionic monoamniotic; *PA* Pulmonary atresia; *PTA* Persistent truncus arteriosus; *PVS* Pulmonary stenosis; *RVOTO* Right ventricular outflow tract obstruction; *VSD* Ventricular septal defect

### Contributions of NCA and pathogenic/likely pathogenic (P/LP) CNV to CHDs

In this study, CMA was performed for all 1118 fetuses, producing a diagnostic yield of 17.6% (197/1118), which included 9.1% (102/1118) for NCA and 8.5% (95/1118) for P/LP CNV.

As expected, NCA was commonly detected in this cohort. The most common was trisomy 18 (3.0%, 33/1118), followed by trisomy 21 (2.7%, 30/1118), trisomy 13 (1.0%, 11/1118), monosomy X (0.7%, 8/1118), mosaic monosomy X (0.4%, 4/1118), mosaic trisomy 13 (0.2% 2/1118), mosaic trisomy 14 (0.2% 2/1118), triploidy (0.2% 2/1118) and others (XYY and other rare mosaic trisomy/monosomy, at one each).

Another common genetic variant observed in the cohort was P/LP CNV. We present the top 19 kinds of CNVs with a frequency of more than once in our cohort in Table 2. The most common was 22q11.21 deletion/duplication with a frequency of 2.5% (28/1118), comprising proximal deletion A-D (including TBX1) (2.1%, 23/1118), central deletion B/C-D (including CRKL) (0.3%, 3/1118), proximal deletion A-B (including TBX1) (n = 1) and proximal duplication A-B (includes TBX1) (n = 1). Another relatively common 16p11.2 deletion/duplication was also detected, with a frequency of 1.0% (11/1118), consisting of proximal deletion BP4-BP5 (including TBX6) (0.9%, 10/1118) and proximal duplication BP4-BP5 (including TBX6) (0.1%, 1/1118). Other recurrently detected P/LP CNVs included 16p13.11 deletion/duplication BP2-BP3 (including MYH11) (0.4%), 2q37.3 terminal deletion (including HDAC4) (0.3%), 4p16.3 terminal deletion (0.3%), and 1q21.2 deletion/duplication (0.3%), among others.

**Table 2:** Pathogenic or likely pathogenic CNVs recurrently occurring in the 1118 fetuses with CHDs

Hot spotsites	Genomic region	Incidence n (%)	CNV (ISCN nomenclature)	Size (Mb)	Origin	Cardiac phenotypes	CNVs associated disorders
22q11.21	Proximal, A-D (includes TBX1)	23 (2.1)	22q11.21(18912231_21465672)×1*	2.55	21 Dn, 1 Pat, 1 Unk	Various types ^§^	22q11.21 proximal deletion syndrome
	Central, B/C-D (includes CRKL)	3 (0.3)	22q11.21(20782219_21915509)×1	1.13	Dn	HLHS+DORV	22q11.21 central deletion syndrome
			22q11.21(21049799_21798907)×1	0.75	Dn	TOF	
			22q11.21(20716876_21465659)×1	0.75	Dn	TOF	
	Proximal, A-B (includes TBX1)	2 (0.2)	22q11.21(18916842_20311858)×1	1.40	Dn	TOF	22q11.21 proximal deletion syndrome
			22q11.1q11.21(16888899_20312661)×3	3.42	Dn	Heterotaxy	22q11.21 proximal duplication syndrome
16p11.2	Proximal, BP4-BP5 (includes TBX6)	11 (1.0)	16p11.2(29649997_30199852)×1*	0.55	8 Dn, 2 Unk	Various types ^&^	16p11.2 proximal microdeletion syndrome
			16p11.2(29649997_30199852)×3	0.55	Dn	PA	16p11.2 proximal microduplication syndrome
	Distal,BP2-BP3 (includes SH2B1)	1 (0.1)	16p11.2(28807417_29051191)×1	0.24	Dn	VSD+COA	16p11.2 distal microdeletion syndrome
16p13.11	BP2-BP3 (include MYH11)	4 (0.4)	16p13.11(15054310_16328840)×3	1.27	Dn	TOF	16p13.11 recurrent microduplication/ microdeletion (neurocognitive disorder susceptibility locus)
			16p13.11p12.3(14892880_16861991)×3	1.97	Unk	COA	
			16p13.11(15481921_16309165)×3	0.83	Unk	VSD+COA	
			16p13.12p13.11(14780543_16525377)×1	1.75	Dn	VSD+COA	
2q37.3	2q37.3 terminal region (includes HDAC4)	3 (0.3)	2q37.1q37.3(234702757_242684920)×1	7.98	Dn	PVS	2q37 microdeletion syndrome
			2q37.2q37.3(237180727_242783384)×1	5.60	Dn	HLHS	
			2q37.3(238892333_242783384)×1	3.89	Dn	Ebstein anomaly	
4p16.3	4p16.3 terminal region	3 (0.3)	4p16.3(1321249_3004266)×1	1.68	Dn	TOF	Wolf-Hirschhorn syndrome
			4p16.3(68345_3950060)×1	3.88	Dn	VSD+COA	
			4p16.3p15.1(68345_29838983)×1	29.77	Dn	VSD+COA	
12p	Isochromosome12p	3 (0.3)	12p13.33p11.1(173786_34788041)×3	34.61	Dn	HLHS	Pallister-Killian syndrome
			12p13.33q12(173786_43520161)×3	43.35	Dn	HLHS	
			12p13.33q13.11(173786_48680002)×3 [0.87]	48.51	Dn	VSD	
1q21.2	distal, BP3-BP4 (includes GJA5)	2 (0.2)	1q21.1q21.2(145382123_147819815)×3	2.44	Unk	PAVPR	1q21.1 duplication syndrome
			1q21.1q21.2(146105170_147897962)×3	1.79	Pat	VSD	
	Proximal, BP2_BP3 (includes RBM8A) and distal, BP3_BP4 (includes GJA5)	1 (0.1)	1q21.1q21.2(145070868_148661862)×1	3.59	Mat	d-TGA	Class II 1q21.1 microdeletion syndrome
7q11.23	7q11.23 recurrent region (includes ELN)	2 (0.2)	7q11.23(72668413_74242132)×1	1.57	Dn	COA	Williams-Beuren syndrome
			7q11.23(72701084_74142190)×1	1.44	Dn	PVS	
8p23.1	8p23.1 recurrentregion (includes GATA4)	2 (0.2)	8p23.1(8093066_11888779)×1	3.80	Dn	VSD	8p23.1 deletion syndrome
			8p23.1(8093066_11935465)×1	3.84	Dn	ASD	
8q24	8q24.12q24.3 ^#^	2 (0.2)	8q24.12q24.3(119261902_146295771)×6-7	27.03	Dn	AVSD	8q24.12-q24.3 segment amplification
			8q24.12q24.3(119328435_146295771)×6–7	26.97	Dn	VSD + ASD	
9p24	9p24.3	2 (0.2)	9p24.3p24.1(208455_7240918)×1[0.6]	7.03	Dn	COA	46,XY sex reversal 4 (9p24.3 deletionsyndrome)
			9p24.3p24.2(203861_4199819)×1	4.00	Dn	HLHS	
11p15	11p15 region (includes H19, KCNQ1)	2 (0.2)	11p15.5p15.4(230615_3413174)×3	3.18	Dn	VSD + COA	Beckwith-Wiedemann syndrome/Silver-Russell syndrome
			11p15.5p15.4(230615_10481292)×3	10.25	Dn	COA	
15q11.2	BP1_BP2 (includesNIPA1)	2 (0.2)	15q11.2(22770421_23283811)×1	0.51	Dn	PVS	15q11.2 deletion syndrome
			15q11.2(22770422_23082328)×1	0.31	Mat	VSD	
17p12	HNPP/CMT1A region (includes PMP22)	2 (0.2)	17p12(14087933_15484858)×1	1.40	Dn	VSD	Hereditary neuropathy with liability to pressure palsies
			17p12(15051374_15882070)×1	0.83	Pat	PAVPR	
18q22-q23	18q22-q23^#^	2 (0.2)	18q22.1q23(65501409_78014123)×3	12.51	Dn	DORV	18q22-q23 duplication
			18q22.2q23(68598182_78014123)×3	9.42	Dn	HLHS	

*Core genomic coordinates of the recurrent CNV region for these cases; ^#^ Not well defined region at present. ^§^ Various types including TOF, PTA, VSD + COA, VSD + IAA, VSD + PTA, PAPVR + ASD,VSD + ASD + IAA + COA, VSD, PA; ^&^ Various types including AVSD, TOF, PVS, PA, VSD, VSD + COA, COA, SV + PVS + d-TGA + PAVPR, Heterotaxy

*AS* Aortic stenosis; *ASD* Atrial septal defect; *AVSD* Atrioventricular septal defect; *CNV* Copy number variant; *COA* Coarctation of aorta; *d-TGA* d-Transposition of great arteries; *Dn* De novo; *DORV* Double outlet right ventricle; *HLHS* Hypoplastic left heart syndrome; *IAA* Interrupted aortic arch; *LVOTO* Left ventricular outflow tract obstruction; *Mat* maternal origin; *PA* Pulmonary atresia; *Pat*, Paternal origin; *PAVPR* Partial anomalous pulmonary venous return; *PTA* Persistent truncus arteriosus; *PVS* Pulmonary stenosis; *RVOTO* Right ventricular outflow tract obstruction; *SV* Single ventricle; *TOF* Tetralogy of Fallot; *Unk* Unknown origin; *VSD* Ventricular septal defect

In addition, CMA detected ROHs in 0.4% (4/1118) of the fetuses, including (5)×2 hmz, 5p15.33p15.32(113577_5240002)×2 hmz (5.13 Mb at 5pter), 19q13.11q13.41(34375323_51784153)×2 hmz (17.41 Mb) and 3p12.3p11.1(78679584_ 90,485,635)×2 hmz (11.81 Mb). The ROH (5)×2 hmz was uniparental disomy (UPD); the other three ROHs were not confirmed as UPD by parental CMA analysis and two of them did not show any P/LP SVs detected by whole-exome sequencing (WES).

### Comparison of NCA and P/LP CNV between isolated and nonisolated CHDs

The frequency of NCA and P/LP CNV in the nonisolated CHD group was significantly higher than that in the isolated CHD group (39.0% vs. 12.1%, *p* < 0.001) (Table 3), which was mainly due to the higher frequency of NCA in the former (nonisolated 27.3% vs. isolated 4.4%, *p* < 0.001). This difference was observed in both singleton and twin fetuses. The frequency of P/LP CNVs in the nonisolated CHD group was higher than that in the isolated CHD group but this difference did not reach significance (11.7% vs. 7.7%, *p* = 0.051). Additionally, the frequency of P/LP CNVs was higher than that of NCA in the isolated CHD group (7.7% vs. 4.4%) but not in the nonisolated CHD group (11.7% vs. 27.3%). This result indicates that P/LP CNVs were more frequently associated with isolated CHDs in this cohort and that NCAs were more frequently associated with nonisolated CHDs. The contributions of NCA and P/LP CNV among various CHD types are presented in Additional file 1: Table S1 and Additional file 2.

**Table 3:** Frequencies of NCA, P/LP CNV and ROH between singleton and twin fetuses with *CHDs*

CMA results		Total (n = 1118)	Isolated (n = 887)	Nonisolated (n = 231)	*p*	Singleton fetuses with CHD				Twin fetuses with CHD				*p* (Singleton vs. Twin)
						Total (n = 961)	Isolated (n = 778)	Nonisolated (n = 183)	*P*	Total (n = 157)	Isolated (n = 109)	Nonisolated (n = 48)	*p*	
CA	NCA (n, %)	102 (9.1)	39 (4.4)	63 (27.3)	< 0.001^*^	80 (8.3)	32 (4.1)	48 (26.2)	< 0.001^*^	22 (14.0)	7 (6.4)	15 (31.3)	< 0.001^*^	0.022^*^
	CNV (n, %)	95 (8.5)	68 (7.7)	27 (11.7)	0.051^*^	83 (8.6)	60 (7.7)	23 (12.6)	0.035^*^	12 (7.6)	8 (7.3)	4 (8.3)	0.829^#^	0.679^*^
	Total (n, %)	197 (17.6)	107 (12.1)	90 (39.0)	< 0.001^*^	163 (17.0)	92 (11.8)	71 (38.8)	< 0.001^*^	34 (21.7)	15 (13.8)	19 (39.6)	< 0.001^*^	0.152^*^
ROH (n, %)	4 (0.4)	2 (0.2)	2 (0.9)	0.191^#^	4 (0.4)	2 (0.3)	2 (1.1)	0.166^#^	0	0	0	–	–	

*CA* Chromosomal abnormalities; *CHD* Congenital heart defect; *CMA* Chromosomal microarray analysis; *CNV* Copy number variant; *NCA* Numerical chromosomal abnormality; *ROH* Region of homozygosity

*Chi-square test; ^#^Fisher's exact test

### Comparison of NCA and P/LP CNV between singleton and twin fetuses

Of interest, the twin group showed a significantly higher frequency of NCA than the singleton group (14.0% vs. 8.3%, *p* = 0.022), but there was no significant difference in the frequency of P/LP CNVs between the groups (7.6% vs. 8.6%, *p* = 0.679) (Table 3). When only taking isolated CHDs into account, the frequency of NCA among twin fetuses was slightly higher than that among singleton fetuses (6.4% vs. 4.1%) but P/LP CNVs were similar (7.3% vs. 7.7%).

In twin fetuses with CHDs, the frequency of NCA was higher than that of P/LP CNV (14.0% vs. 7.6%); in particular, the frequency of NCA reached to 31.3% (15/48) in twin fetuses with nonisolated CHDs. However, when only considering isolated CHDs, the contribution of NCA and P/LP CNVs to CHDs showed little difference.

### Contribution of P/LP SVs in fetuses without NCAs and P/LP CNVs

For the 921 fetuses without NCAs and P/LP CNVs (including 917 fetuses with negative CMA results and 4 fetuses with ROHs detected by CMA), fetus-only WES or fetus-parent WES (trio-WES) was conducted for further investigation in 62 fetuses (including 59 fetuses from singleton pregnancies and 3 fetuses from 3 twin pregnancies): 41 with isolated CHDs and 21 with nonisolated CHDs. Compared with CMA, WES produced an incremental diagnostic yield of 12.9% (8/62) for P/LP SVs. These P/LP SVs were detected in 8 fetuses from singleton pregnancies. These SVs, consisting of 5 frameshift variants, 1 splicing variant and 2 missense variants, were detected in 8 genes known to be associated with CHD, including KMT2D, WAC, CHD7, RAF1, EP300, GDF1, PQBP1 and LZTR1 (Table 4).

**Table 4:** Pathogenic or likely pathogenic sequence variants identified in the 62 fetuses with CHDs

Case	Cardiac defects	Extracardiac malformations	Gene	Sequence variant	Genotype	Origin	Classification	Disorders (OMIM number; Mode of inheritance)	Reported variant	Outcome
1	HLHS	Hydronephrosis, renal agenesis	KMT2D	NM_003482.4:c.5485dup(p.Ile1829Asnfs*4)	Het	Dn	P	Kabuki syndrome 1 (147,920; AD)	No	TOP
2	HLHS	–	WAC	NM_016628.5:c.1394delT(Ile465Thrfs*4)	Het	Dn	P	Desanto-Shinawi syndrome (616,708; AD)	No	TOP
3	d-TGA, DORV, VSD, ASD	–	CHD7	NM_017780.4: c.3018delT (p.Leu1007Serfs*6)	Het	Dn	P	CHARGE syndrome (214,800; AD); Hypogonadotropic hypogonadism 5 with or without anosmia (612,370; AD)	No	TOP
4	VSD	–	RAF1	NM_002880.4: c.1082G > C (p.Gly361Ala)	Het	Dn	P	Noonan syndrome 5 (611,553; AD); LEOPARD syndrome 2 (611,554; Un); Cardiomyopathy, dilated, 1NN (615,916; AD)	Yes	TOP
5	VSD + IAA	Hydrocephalus, hypospadias	EP300	NM_001429.4:c.3671 + 1G > A	Het	Dn	LP	Menke-Hennekam syndrome 2 (618,333; AD), Rubinstein-Taybi syndrome 2 (613,684; AD)	Yes	TOP
6	AVSD	–	GDF1	NM_001492.6:c.996delC(p.Asp333Thrfs*52)	Het	Dn	LP	Congenital heart defects, multiple types, 6 (613,854; AD)	No	TOP
7	DORV, VSD	FGR, facial flatness, suspected craniosynostosis	PQBP1	NM_001032382.2: c.459_462del (p.Arg153Serfs*41)	Hemi	Mat	P	Renpenning syndrome (309,500; XLR)	Yes	TOP
8	VSD, COA	-	LZTR1	NM_006767.4: c.851G > A (p.Arg284His)	Het	Pat	LP	Noonan syndrome 10 (616,564; AD); Noonan syndrome 2 (605,275; AR)	Yes	TOP
			SOS2	NM_006939.4:c.708A > T(p.Lys236Asn)	Het	Mat	VUS	Noonan syndrome 9 (616,559; AD)	No	

*AD* Autosomal dominant; *AR* Autosomal recessive; *ASD* Atrial septal defect; *AVSD* Atrioventricular septal defect; *COA* Coarctation of aorta; *d-TGA* d-Transposition of great arteries; *Dn* de novo; *DORV* Double outlet right ventricle; *Dup* Duplication; *FGR* Fetal growth restriction; *Hemi* Hemizygous; *Het* Heterozygous; *HLHS* Hypoplastic left heart syndrome; *LP* Likely pathogenic; *Mat* Maternal origin; *P* Pathogenic; *PA* Pulmonary atresia; *PLSVC* Persistent left superior vena cava; *TOP* Termination of pregnancy; *VSD* Ventricular septal defect; *VUS* Variant of uncertain significance; *XLR* X-linked recessive

This study identified 8 P/LP SVs, 4 of which are novel variants and the others are known variants reported previously. Six of the eight variants are de novo. However, for two parental inherited P/LP SVs in two affected fetuses in this study, one is an X linked recessive PQBP1 gene, and it is understandable that the maternal inherited PQBP1 variant resulted in CHDs in a male fetus; however, another variant with an autosomal dominant inheritance in the LZTR1 gene (c.851G > A, p.Arg284His) was identified in both the affected fetus and healthy father, which led to a prenatal counseling dilemmas, although the variant was classified as LP variants according to American College of Medical Genetics and Genomics (ACMG) and Clinical Genome Resource consensus recommendation (ClinGen) guidelines (Additional files 2 and 3). This kind of phenomenon is usually attributed to incomplete penetrance, which is not uncommon in CHD [10, 13]. The prenatal counseling dilemmas is due to the fact that the cause of incomplete penetrance for this kind of variant is not currently known, and understanding the genotype–phenotype association is difficult.

### Hotspot P/LP CNVs contributing to CHDs

A total of 109 P/LP CNVs were identified in 95 (8.5%) fetuses with CHDs, of which 97 (89.0%) were de novo CNVs and 6 (5.5%) parental inherited CNVs; 6 (5.5%) had unknown parental information. The two most common P/LP CNVs were 22q11.21 deletion/duplication (2.5%, 28/1118) and 16p11.2 deletion/duplication (1.0%, 11/1118); the former showed a frequency of 92.9% (26/28) for de novo events, and the latter showed a frequency of 81.8% (9/11) (Table 2). More specifically, a de novo frequency of 91.3% (21/23) was observed for the 22q11.21 proximal deletion A-D (including TBX1); the 16p11.2 proximal deletion BP4-BP5 (including TBX6) showed 80% (8/10). Table 5 presents ultrasound findings, clinical characteristics and CNV descriptions for the 11 CHD fetuses carrying 16p11.2 deletion (n = 10) or duplication (n = 1).

**Table 5:** Ultrasound findings, clinical characteristics and CNV descriptions for the 11 CHD fetuses carrying 16p11.2 recurrent deletion or duplication

Case	Maternal age (years)	History	Gestation at diagnosis (weeks)	Cardiac defects	Extracardiac malformations	Soft markers	CNV state	Genomic coordinates	Size (Mb)	Origin	Outcome
1	24	G1P0	26	AVSD	Findings after birth: congenital hypothyroidism	NF	Del	29,591,078–30177916	0.59	Dn	Live birth
2	35	G4P2	32	ASD, COA	-	PLSVC	Del	29,567,295–30,178,406	0.61	Dn	Live birth
3	28	G2P1	26	VSD, COA	Findings after birth: Hand polydactyly (left), absence of metacarpophalangeal joint (left), talipes equinovarus (left)	-	Del	29,432,212–30,240,227	0.81	Unk	Live birth
4	38	G2P1	25	PVS	–	–	Del	29,567,295–30,177,999	0.61	Dn	TOP
5	31	G3P1	25	TOF	–	RAA	Del	28,340,569–30177999	1.84	Dn	TOP
6	35	G2P1	21	AVSD	Findings after birth: Hirschsprung, Gastric pyloric stenosis	–	Del	29,567,295–30,190,029	0.62	Dn	Live birth
7	29	G1P0	23	SV, PVS, d-TGA, PAVPR	–	–	Del	29,567,296–30,190,029	0.62	Dn	TOP
8	29	G2P0	24	VSD	FGR	PLSVC	Del	29,428,531–30,190,029	0.76	Unk	Live birth
9	25	G1P0	22	PA	FGR, hydropericardium	–	Del	29,342,193–30,350,748	1.01	Dn	TOP
10	34	G2P1	23	Dextrocardia, ASD, VSD, PVS, DORV	Asplenia, abnormal position of gastric vesicles and liver	–	Del	29,591,327–30,190,029	0.60	Dn	TOP
11	36	G1P0	19	PA	–	–	Dup	29,597,823–30,177,240	0.58	Dn	TOP

*ASD* Atrial septal defect; *AVSD* Atrioventricular septal defect; *CNV* Copy number variant; *COA* Coarctation of aorta; *d-TGA* d-Transposition of great arteries; *Del* Deletion; *Dn* de novo; *DORV* Double outlet right ventricle; *Dup* Duplication; *FGR* Fetal growth restriction; *NF* Thickened nuchal fold; *PA* Pulmonary atresia; *pat* Paternal origin; *PAVPR* Partial anomalous pulmonary venous return; *PLSVC* Persistent left superior vena cava; *PVS* Pulmonary stenosis; *RAA* Right aortic arch; *SV* Single ventricle; *TOF* Tetralogy of Fallot; *TOP* Termination of pregnancy; *Unk* Unknown origin; *VSD* Ventricular septal defect

We considered the 16p11.2 proximal deletion BP4-BP5 (including TBX6) as a potential hotspot CNV for CHD. First, most of the 16p11.2 deletions in our study are de novo occurrences consistent with the perspectives of previous studies that genetic variants originating from de novo events confer a critical contribution to CHD [9, 10, 13]. The deletion has been reported sporadically in other previous cohorts, case reports or the DECIPHER database (ID: 251,630, 278,277, 288,280, 357,605, 359,216) [15, 17, 19–21, 23–29], but it was repeatedly observed in our large cohort. Second, comparison of the frequency of the deletion in our cohort with that in a previous control cohort (6/22246) indicated apparent enrichment of the deletion (*p* < 0.001) in our fetal CHD cohort [30]. Third, WES analysis performed for the ten fetuses carrying the deletion excluded causative P/LP SVs or other cardiovascular phenotype-associated rare variants of functionally intolerant genes considered to be modifying factors for interpretation of incomplete penetrance and variable expressivity of CNV [31].

## Discussion

Although there have been some prenatal cohort studies on associations between genetic variants and CHDs [16, 17, 19–21, 32], to the best of our knowledge, this study is the largest cohort to investigate the contribution of genetic variants to CHDs in fetuses in southern China. The contributions of the diverse genetic variants mentioned above in this study include some few differences from previous studies [16, 19–21], but our cohort recruited fetuses with CHDs of both singleton pregnancies and twin pregnancies and this might be expected to be highly representative of the current practice of prenatal diagnosis. This work mainly shows that NCA and P/LP CNV are important genetic variants contributing to CHDs in fetuses, most of which are de novo variants. We also present a variety of recurrent CNVs related to CHDs in fetuses. These CNVs may be worthy of further study to investigate their pathogenic mechanism in CHD.

This study confirmed that the 16p11.2 deletion is a hotspot CHD-associated CNV (explained in Results section). The deletion is one of the most common CNVs for NDDs (such as autism spectrum disorder) widely identified in postnatal cases and less commonly recognized in cases with congenital anomalies involving the spine, kidney and urinary tract and brain, rarely garnering intensive attention in CHDs [24, 33–38]. The high frequency of the deletion has not been revealed in previous prenatal cohorts but only reported in a postnatal study [24]. This discordance may be attributed to the inclusion criteria of case types (pre- or postnatal cases), sample sizes, CHD types, CHD concomitant extracardiac anomaly types or region and population differences. Of note, NDDs frequently occur in patients with CHDs, in 10% of patients with CHDs and in 50% of patients with severe CHDs [7]. For example, for the 16p11.2 deletion, the penetrance of CHD phenotypes in individuals with the deletion is estimated to be no more than 10% in postnatal cohorts [24, 39]. Our previous study also showed that CHDs are not uncommon in fetuses (4/12) carrying 16p11.2 deletions [38]. However, the penetrance of 16p11.2 deletion-associated NDDs is much higher than that of CHDs [24]. Identification of the 16p11.2 deletion in fetuses regardless of mild or severe CHDs would be a potential reminder of NDDs and have significant value for prenatal diagnosis and genetic counseling. In general, medical management and prognosis between CHDs with P/LP CNV and CHDs without P/LP CNV are apparently different, as demonstrated by a study on the 22q11.2 deletion [40].

In addition, several studies with either relatively small or large sample sizes have reported an incremental yield of P/LP SVs ranging from 4.5 to 12.7% in CHD fetuses without NCAs and CNVs [17–21, 41]. Although a limited number of patients underwent WES in our study, WES also showed an incremental yield of 12.9% for P/LP SVs. Importantly, de novo P/LP SVs with autosomal dominant inheritance are the main type of causative variants in this study. This finding is consistent with previous studies showing that ~ 80% of P/LP SVs contributing to CHDs originate from de novo occurrence [8, 10, 18].

Furthermore, previous CHD cohort studies included only singleton fetuses but not twin fetuses [16, 17, 19–21, 32]. For twin pregnancies, although cardiac hemodynamic changes from vascular anastomoses in the placenta and some influence from the special intrauterine environment of twins might have a secondary impact on fetal cardiovascular morphogenesis, the contributions cannot be quantitatively estimated [42]. Nonetheless, previous studies have indicated that a portion of CHDs in twin fetuses result from NCA and P/LP CNV [43, 44]. Our study also reveals that approximately 21.7% of CHD in twin fetuses result from NCA and CNV, with subtle differences between singleton and twin fetuses regarding the contributions of NCA and CNV, as mentioned in Table 3.

However, the primary limitation of our study is the small sample size of fetuses who underwent WES, limiting deep insight into the associations between SVs and CHDs. It was also not possible to estimate the cost-effectiveness and clinical performance of whether CMA and WES should be performed in parallel or sequentially, as prenatal genetic testing strategies are worthy of attention in the current genomic era.

Currently, definitive causal genetic variants contributing to CHDs have been identified in no more than 40% of patients with CHD [14]. In addition, the mean rate of CHD recurrence is approximately 3.1% in the offspring of patients with CHDs [45], indicating that de novo variants only explain a proportion of CHDs. In general, increased understanding of genotype-CHD phenotypes has led to new insight into the molecular pathogenesis of CHDs, and other genetic variants, such as oligogenic or polygenic risk factors, noncoding variants and structural variants, have been recognized as potential mechanisms for CHD cases lacking definitive causes. We believe that accumulating comprehensive whole-genome sequencing genotype and phenotype datasets is essential to exploring the residual etiology of CHDs, which will improve our understanding of the contributions of currently undefined genetic variants to the pathogenesis.

## Conclusions

In conclusion, our cohort study provides a deep profile of the contribution of genetic variants to CHDs in both singleton and twin fetuses; NCA and P/LP CNV contribute to 9.1% and 8.5% of CHD in fetuses, respectively. Furthermore, we confirmed the 16p11.2 deletion as a CHD-associated hotspot CNV, second only to the 22q11.21 deletion in frequency. Most 16p11.2 deletions detected were de novo. Additionally, P/LP SV was identified in 12.9% (8/62) of fetuses without NCA or P/LP CNV.

## Methods

### Subjects

This study reviewed singleton and twin pregnancies with fetal CHD registered in prenatal diagnosis databases of the First Affiliated Hospital of Sun Yat-sen University, the First Affiliated Hospital of Jinan University and Guangdong Women and Children Hospital from March 2016 to June 2022. The inclusion criteria were as follows: (1) Prenatal ultrasound examination, echocardiography and follow-up information were available; (2) Fetuses were diagnosed with CHD by prenatal ultrasound examination and echocardiography; (3) Fetuses with confirmed CHD underwent CMA prenatally; (4) for some fetuses with negative CMA results or ROH, prenatal WES was offered based on parents’ informed consent and willingness. The exclusion criteria included the following: (1) Prenatal follow-up ultrasound examination or echocardiography excluding a diagnosis of CHDs; (2) Isolated cardiovascular ultrasound soft markers or common structural variations including isolated right aortic arch, persistent left superior vena cava, aberrant right subclavian artery, left venous catheter and atrial septal aneurysm; (3) Monochorionic twins with specific complications including twin-to-twin transfusion syndrome, selective intrauterine growth restriction, twin reversed arterial perfusion sequence, twin anemia-polycythemia sequence and conjoined twins.

CHDs were classified as isolated or nonisolated CHDs. The latter included CHDs with one or multiple ECMs, fetal growth restriction or amniotic fluid volume abnormalities.

In all cases, consultation with both a genetic counselor and a prenatal diagnostician was carried out before invasive prenatal puncture sampling, genetic testing and other medical management. Fetal specimens included amniotic fluid or umbilical cord blood. This study was approved by the Medicine Ethical Committee of three units, and the parents of all fetuses provided written informed consent for prenatal puncture sampling and genetic testing.

### Chromosomal microarray analysis

Genomic DNA was extracted from amniotic fluid, umbilical cord blood or tissues of induction labor with a QIAamp DNA Blood Mini kit (Qiagen, Germany). CNVs and regions of homozygosity (ROHs) were detected using a CytoScan HD or 750K chip on the single-nucleotide polymorphism array platform according to the manufacturer's standard operating procedures (Thermo Fisher Scientific, USA). Chromosome Analysis Suite software (Thermo Fisher Scientific, USA) was applied to analyze data based on genome version GRCh37/hg19. A threshold of resolution at least 100 kb called by ≥ 50 contiguous probes for CNV and at least 5 Mb for ROH was established. CNV and ROH were classified according to the ACMG and ClinGen guidelines. Pathogenic (P) and likely pathogenic (LP) CNVs are abbreviated to P/LP CNVs. In this study, the scope of CNVs includes deletion/duplication of fragments ≥ 100 kb in size and chromosomal segment deletion/duplication of fragments ≥ 10 Mb in size; numerical chromosomal abnormality (NCA) refers to aneuploidy, polyploidy and mosaic NCA.

### Whole-exome sequencing

Using genomic DNA extracted from amniotic fluid, umbilical cord blood or tissues of induction labor with a QIAamp DNA Blood Mini kit (Qiagen, USA), exome sequences were captured with an Agilent SureSelect Human All Exon capture kit v6 (Agilent, USA). Genomic DNA was fragmented randomly, purified, and enriched to construct DNA libraries and then sequenced using the NextSeq500 platform according to the manufacturer's protocols (Illumina, USA). The sequencing reads were aligned to the reference genome sequence (GRCh37/hg19) using BWA software. After alignment, SAMtools software was used to create, sort, and index bam files. Duplicate reads and multiple mapped reads in the exome were removed using Picard software. Calling and annotation of single-nucleotide variants and small insertions/deletions were performed using GATK and ANNOVAR software, respectively. Data quality control criteria reached to an average sequencing depth of ≥ 150× and a minimum coverage of 20× for ≥ 98% of targeted regions. Variant filtering and selection mainly utilized the following criteria: variants with sequencing depth ≥ 20 and alternate allele proportion ≥ 0.3; variants in known disease-causing genes; absent or rare variants (minor allele frequency < 0.01); deleterious variants as predicted by computational prediction tools; variants fulfilling disease inheritance models or family cosegregation; and variants with previously reported cases and/or supported by experimental evidence through searching public literature and databases. The minor allele frequencies of all detected variants were determined according to their frequencies in public population databases, including the gnomAD, dbSNP, 1000 Genomes Project and ESP6500 databases. Computational prediction tools, including SIFT, PolyPhen-2, Mutation Taster, PROVEAN, CADD, Revel, SpliceAI and MaxEntScan, were used to predict whether a variant had a deleterious effect on the gene. Databases such as OMIM, ClinVar, HGMD, LOVD and PubMed were used to assist in the interpretation of variant pathogenicity. Variant pathogenicity classification followed ACMG and ClinGen guidelines. P/LP SVs associated with CHDs are considered causative SVs.

Prenatal WES was performed for some fetuses with negative CMA results (including 59 fetuses from singleton pregnancies and 3 fetuses from 3 twin pregnancies) to detect causative P/LP SVs.

In addition, because 16p11.2 proximal deletion BP4-BP5 (including TBX6) was less frequently identified in cases with CHDs and their correlation was not reliably confirmed in previous studies, prenatal WES was also performed for fetuses carrying the 16p11.2 deletion detected in this study to exclude causative P/LP SVs and cardiovascular phenotype-associated deleteriously rare variants in functionally intolerant genes (“other hits”) [31].

### Statistical analysis

SPSS statistics software (IBM SPSS statistics version 22.0) was used for statistical analysis. The statistical analysis methods performed included the chi-square test or Fisher’s exact test with *p* < 0.05 considered statistically significant.

### Supplementary Information

**Additional file 1.** The frequencies of NCA, P/LP CNV and ROH among different types of CHD.

**Additional file 2.** Contributions of NCA and P/LP CNV among various CHD types and interpretation for classification of LZTR1 variant.

**Additional file 3.** Homology analysis of LZTR1 proteins among various species (**A**) and protein structural models of LZTR1 with the Arg284His variant (**B**).

## Data Availability

The datasets used and/or analysed during the current study are available from the corresponding author on reasonable request.

## Abbreviations

CHD: Congenital heart defect
CMA: Chromosomal microarray analysis
CNV: Copy number variant
ECM: Extracardiac malformation
LP: Likely pathogenic
NDD: Neurodevelopmental disorder
P: Pathogenic
ROH: Region of homozygosity
SV: Sequence variant
WES: Whole-exome sequencing
