# Supplementary material for: Contribution of genetic variants to congenital heart defects in both singleton and twin fetuses: a Chinese cohort study
Source: Mol Cytogenet. 2024 Jan 4;17:2. doi: 10.1186/s13039-023-00664-y (PMC10768341; doi:10.1186/s13039-023-00664-y)
Supplement: Supplementary file 1 — Additional file 1. The frequencies of NCA, P/LP CNV and ROH among different types of CHD. [file 13039_2023_664_MOESM1_ESM.docx]

**Contribution of genetic variants to congenital heart defects in both singleton and twin fetuses: a Chinese cohort study**

**Supplemental Results**

**Contributions of NCA and P/LP CNV among various CHD types**

These results are summarized in Additional file 1: Table S1. The most common CHD types were septal defects (32.1%, 359/1118) and conotruncal defects (25.4%, 284/1118), accounting for 57.5% of CHDs diagnosed prenatally. The frequency of NCA and P/LP CNV was 18.7% and 15.5% for the two CHD types, respectively. Among other less common CHD types, the frequency of NCA and P/LP CNV was also high in AVSD (28.6%), single ventricle/complex (28.2%) and LVOTO (20.2%) but relatively lower in heterotaxy (12.5%), RVOTO (11.3%) and other types (2.9%).

For the isolated CHD group, we noted that the frequency of P/LP CNV was lower than that of NCA in septal defects (3.3% vs. 7.0%), AVSD (7.5% vs. 10.0%) and single ventricle/complex (7.7% vs. 15.4%). In contrast, the frequency of P/LP CNV was higher than that of NCA in conotruncal defects (9.0% vs. 2.9%), RVOTO (7.3% vs. 0), LVOTO (10.4% vs. 5.2%), heterotaxy (7.7% vs. 0), APVR (25.0% vs. 0) and associations (14.3% vs. 2.0%).

**Interpretation for classification of LZTR1 variant**

The LZTR1 variant (c.851G>A, p.Arg284His) was identified in both the affected fetus and healthy father. The amino acid Arg284His is located in the Kelch motif and is highly conserved among various species (Additional file 3: Fig.S1-A). Protein structural models of LZTR1 with the Arg284His variant showed that it breaks a salt bridge formed in the wild-type protein, which is buried and leads to expansion or contraction of the cavity volume, indicating that it has important impacts on the stability and function of the LZTR1 protein (Additional file 3: Fig.S1-B). The variant has not been reported in patients with Noonan syndrome, but another variant affecting the same amino acid c.850C>T(p.Arg284Cys) has been observed in individuals with Noonan syndrome [1]. Therefore, the variant is recognized as a likely pathogenic variant according to ACMG and ClinGen guidelines (PP3_Strong+PM5+PM2_Supporting).

**References**

1. Jacquinet A, Bonnard A, Capri Y, Martin D, Sadzot B, Bianchi E, et al. Oligo-astrocytoma in LZTR1-related Noonan syndrome. Eur J Med Genet. 2020;63(1):103617.
