# Supplementary material for: Contribution of genetic variants to congenital heart defects in both singleton and twin fetuses: a Chinese cohort study
Source: Mol Cytogenet. 2024 Jan 4;17:2. doi: 10.1186/s13039-023-00664-y (PMC10768341; doi:10.1186/s13039-023-00664-y)
Supplement: Supplementary file 2 — Additional file 2. Contributions of NCA and P/LP CNV among various CHD types and interpretation for classification of LZTR1 variant. [file 13039_2023_664_MOESM2_ESM.docx]

| **Supplemental Table S1** Frequencies of NCA, P/LP CNV and ROH among different types of CHD. | | | | | | | | | | | | | | |
| --- | --- | --- | --- | --- | --- | --- | --- | --- | --- | --- | --- | --- | --- | --- |
| CHD types | **Isolated**  **n (%)** | | | | | **Nonisolated**  **n (%)** | | | | | **Total**  **n (%)** | | | |
|  | N | CA (NCA+CNV) | NCA | CNV | ROH | N | CA (NCA+CNV) | NCA | CNV | ROH | N | CA (NCA+CNV) | NCA | CNV |
| Septal Defects | 270 | 28 (10.4) | 19 (7.0) | 9 (3.3) | 0 | 89 | 39 (43.8) | 33 (37.1) | 6 (6.7) | 1 (1.1) | 359 | 67 (18.7) | 52 (14.5) | 15 (4.2) |
| Conotruncal defects | 244 | 29 (11.9) | 7 (2.9) | 22 (9.0) | 0 | 40 | 15 (37.5) | 10 (25.0) | 5 (12.5) | 0 | 284 | 44 (15.5) | 17 (6.0) | 27 (9.5) |
| RVOTO | 96 | 7 (7.3) | 0 | 7 (7.3） | 0 | 10 | 5 (71.4) | 0 | 5 (71.4) | 0 | 106 | 12 (11.3) | 0 | 12 (11.3) |
| LVOTO | 58 | 9 (15.5) | 3 (5.2) | 6 (10.4) | 0 | 31 | 9 (29.0) | 7 (22.6) | 2 (6.4) | 1 (3.2) | 89 | 18 (20.2) | 10 (11.2) | 8 (9.0) |
| AVSD | 40 | 7 (17.5) | 4 (10.0) | 3 (7.5) | 0 | 9 | 7 (77.8) | 5 (55.6) | 2 (22.2) | 0 | 49 | 14 (28.6) | 9 (18.4) | 5 (10.2) |
| Heterotaxy | 13 | 1 (7.7) | 0 | 1 (7.7) | 0 | 19 | 3 (15.8) | 1 (5.3) | 2 (10.5) | 0 | 32 | 4 (12.5) | 1 (3.1) | 3 (9.4) |
| APVR | 12 | 3 (25.0) | 0 | 3 (25.0) | 0 | 0 | 0 | 0 | 0 | 0 | 12 | 3 (25.0) | 0 | 3 (25.0) |
| Associations | 98 | 16 (16.3) | 2 (2.0) | 14 (14.3) | 1 (1.0) | 16 | 7 (43.8) | 4 (25.0) | 3 (18.8) | 0 | 114 | 23 (20.2) | 6 (5.3) | 17 (14.9) |
| Single Ventricle  /Complex | 26 | 6 (23.1) | 4 (15.4) | 2 (7.7) | 0 | 13 | 5 (38.5) | 3 (23.1) | 2 (15.4) | 0 | 39 | 11 (28.2) | 7 (17.9) | 4 (10.3) |
| Others | 30 | 1 (3.3) | 0 | 1 (3.3) | 1 (3.3) | 4 | 0 | 0 | 0 | 0 | 34 | 1 (2.9) | 0 | 1 (2.9) |
| AVSD, atrioventricular septal defect; APVR, anomalous-pulmonary venous return; CA, chromosomal abnormalities; CNV, copy number variant; LVOTO, left ventricular outflow tract obstruction; NCA, numerical chromosomal abnormality; ROH, region of homozygosity; RVOTO, right ventricular outflow tract obstruction. | | | | | | | | | | | | | | |
