# Supplementary figures and images for: Contribution of genetic variants to congenital heart defects in both singleton and twin fetuses: a Chinese cohort study
Source: Mol Cytogenet. 2024 Jan 4;17:2. doi: 10.1186/s13039-023-00664-y (PMC10768341; doi:10.1186/s13039-023-00664-y)

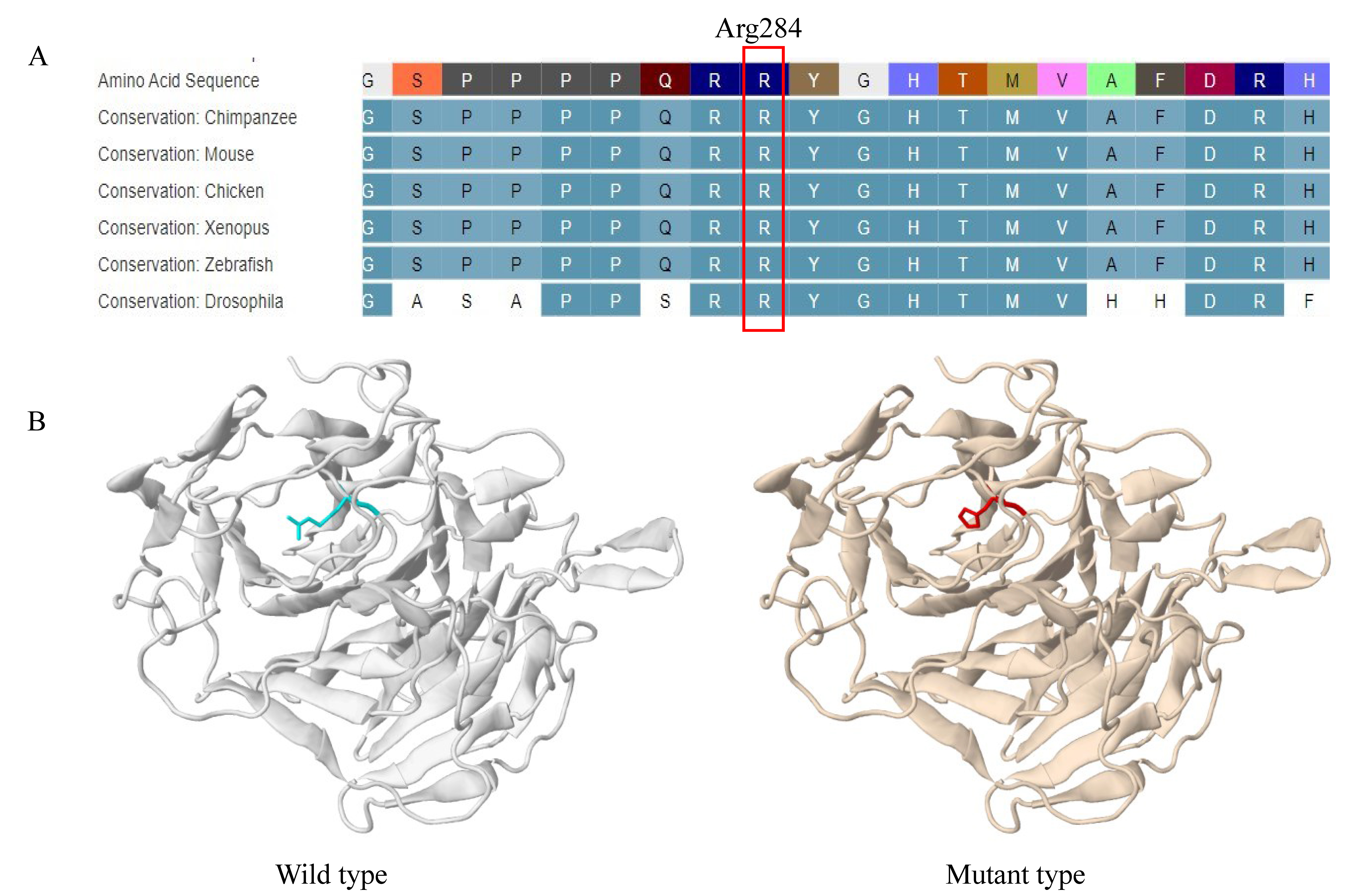

Supplement: Supplementary file 3 — Additional file 3. Homology analysis of LZTR1 proteins among various species (A) and protein structural models of LZTR1 with the Arg284His variant (B). [file 13039_2023_664_MOESM3_ESM.jpg]
